# Role of Ion Channels in the Maintenance of Sperm Motility and Swimming Behavior in a Marine Teleost

**DOI:** 10.3390/ijms232012113

**Published:** 2022-10-11

**Authors:** Júlia Castro-Arnau, François Chauvigné, Joan Cerdà

**Affiliations:** 1 Institute of Agrifood Research and Technology (IRTA)-Institute of Biotechnology and Biomedicine (IBB), Universitat Autònoma de Barcelona, 08193 Barcelona, Spain; 2 Institute of Marine Sciences, Spanish National Research Council (CSIC), 08003 Barcelona, Spain

**Keywords:** spermatozoa, ions, pH, activation, transcriptome, ion channels, motility, trajectory

## Abstract

In oviparous marine fishes, the hyperosmotic induction of sperm motility in seawater (SW) is well established, however, the potential function of ion channels in the maintenance of post activated spermatozoon swimming performance remains largely unknown. Here, we investigated the influence of ion channels on the spermatozoon swimming parameters using the gilthead seabream (*Sparus aurata*) as a model for modern marine teleosts. Our data show that the SW-induced activation of seabream sperm motility requires three concomitant processes, the hyperosmotic shock, an ion-flux independent increase of the intracellular concentration of Ca^2+^ ([Ca^2+^]_i_), but not of [K^+^]_i_ or [Na^+^]_i_, and the alkalization of the cytosol. The combination of all three processes is obligatory to trigger flagellar beating. However, the time-course monitoring of sperm motion kinetics and changes in the [Ca^2+^]_i_, [K^+^]_i_ and [Na^+^]_i_ in SW or in non-ionic activation media, showed that the post activated maintenance of spermatozoa motility is dependent on extracellular Ca^2+^ and K^+^. A meta-analysis of a seabream sperm transcriptome uncovered the expression of multiple ion channels, some of which were immunolocalized in the head and/or tail of the spermatozoon. Selective pharmacological inhibition of these ion channel families impaired the long-term motility, progressivity, and velocity of SW-activated spermatozoa. The data further revealed that some antagonists of K^+^-selective or Ca^2+^-selective channels, as well as of stretch-activated and mechanosensitive channels, altered the trajectory of spermatozoa, suggesting that these ion channels are likely involved in the control of the swimming pattern of the post activated spermatozoon. These combined findings provide new insight into the signaling pathways regulating spermatozoon activation and swimming performance in marine fishes.

## 1. Introduction

The activation of motility and maintenance of spermatozoon swimming performance are crucial for successful fertilization in vertebrates. However, the two processes occur in entirely different spatial compartments or ionic milieus, depending on whether fertilization is internal or external. For example, in internally fertilizing mammals, sperm released from the epididymis is hyperactivated in the confined hypotonic compartments of the female oviduct [1]. Conversely, in oviparous fish, spermatozoa remain quiescent in the testes and efferent ducts and the initiation of motility is triggered by the hypo- or hyperosmotic aquatic environment into which the sperm are ejaculated [2].

In mammals and most fish species, a common signaling event for the activation of flagellar beating and spermatozoa swimming is an increase of the intracellular Ca^2+^ concentration ([Ca^2+^]_i_) [3,4]. However, the spermatozoa of fish and mammals face entirely different ionic environments following ejaculation. The [K^+^] and [Na^+^] in freshwater (FW) are extremely low (0.1 to 0.7 mM) compared to the orders-of-magnitude higher concentrations in seawater (SW) or the oviduct [5]. Furthermore, [Ca^2+^] in SW is much higher (10 mM) than in FW (<1 mM). Due to such differences in the ionic activation milieu, mammalian and piscine spermatozoa are thought to have evolved specific molecular mechanisms that differentially control Ca^2+^ signaling [6].

In mammalian sperm, Ca^2+^ influx is mediated by a sperm-specific, voltage-dependent Ca^2+^ channel complex called CatSper. CatSpers open in response to a transient membrane hyperpolarization and further alkalization of the intracellular pH (pH_i_) that is induced by Na^+^/H^+^ exchangers (NHEs), HCO_3_^−^ and monocarboxylate membrane transporters, or voltage-gated H^+^ transporters, or by direct stimulation with prostaglandins and progesterone in the seminal fluid or the oviduct [7,8]. In FW fishes, the initiation of spermatozoon motility is regulated by the external hypo-osmolality leading to K^+^ efflux via K^+^-selective cyclic nucleotide-gated (CNGK) channels and a consequent hyperpolarization of the membrane potential [5]. The activation process may also involve stretch-activated ion channels (SACs), which augments the [Ca^2+^]_i_ via Ca^2+^ channels, and the pH_i_ through the activation of NHEs as in mammals [2,9]. Subsequently, axonemal beating is likely triggered by protein kinase C and other yet unknown Ca^2+^-calmodulin-dependent proteins in the axoneme [2]. A recent study on zebrafish (*Danio rerio*) suggests, however, that the hyperpolarization mediated by CNGK, which is not activated by cyclic nucleotides but by the alkalization of the pH_i_ [5], is also regulated by a highly H^+^-selective voltage-gated “pacemaker” channel (Hcnl1), which depolarizes the membrane potential and closes CNGK channels by intracellular acidification, and therefore both channels appear to be functionally linked [10].

In some FW fishes, however, such as salmonids and sturgeons, the major trigger of sperm motility may not be osmolarity but the decrease in [K^+^] in FW with respect to that in the seminal fluid within the efferent duct, which causes K^+^ efflux through yet unknown K^+^ channels that hyperpolarize the sperm membrane [2]. This, in turn, opens L-type or T-type Ca^2+^ channels, and perhaps also orthologs of the mammalian CatSper (salmonids), that together with the mobilization of intracellular Ca^2+^ stores, raise the [Ca^2+^]_i_, and activate the production of cAMP by adenylyl cyclase, which induces the phosphorylation of protein kinase A and axonemal proteins [2,9,11]. In sturgeons, the increase of the [Ca^2+^]_i_ may be independent of Ca^2+^ influx and induces motility via Ca^2+^/calmodulin activated phosphodiesterases [2].

In most marine teleosts, sperm motility is triggered by exposure to the hyperosmotic SW and requires a surge of the [Ca^2+^]_i_, and in some cases also of [K^+^]_i_, which may be associated with the alkalization of the pH_i_ [2,3,9,12]. These events activate the axonemal machinery directly, or through the Ca^2+^/calmodulin- or cAMP-dependent and -independent protein phosphorylation/dephosphorylation of axonemal proteins [2,13]. However, the mechanisms that mediate Ca^2+^ and K^+^ signaling appear to be species-specific. In some species, Ca^2+^ influx during ligand-induced motility initiation may occur via reverse-Na^+^/Ca^2+^ exchangers or CatSper, whereas in others the increase of [Ca^2+^]_i_ and/or [K^+^]_i_ may be the result of the stimulation of SACs in response to osmotic or mechanical changes [2,9,14]. In other cases, such in the gilthead seabream (*Sparus aurata*), the rise of [Ca^2+^]_i_ and [K^+^]_i_ in the spermatozoon is independent of extracellular ions, and occurs from the increased cytosolic concentration resulting from the massive water efflux mediated by flagellar aquaporin-1aa (Aqp1aa) following the hyperosmotic shock, and/or by Ca^2+^ release from intracellular stores [2,13,15].

In contrast to the mechanisms involved in motility activation, the roles of ions and ion channels in the maintenance of the velocity and swimming behavior of post activated fish spermatozoa are much less known. Such mechanisms are nevertheless hypothesized to play important roles in physical and chemoattractant signaling pathways that guide and orient the spermatozoa towards the egg [16,17,18,19]. For example, a recent study in zebrafish suggests that the functional interaction of CNGK and Hcnl1 channels, both located in the spermatozoon head, may be crucial for successful navigation to the egg micropyle [5,10]. In addition, the activation of a thermosensitive Ca^2+^ channel (transient receptor potential cation channel subfamily V member 1; Trpv1), that is expressed in the head, mid piece, and tail regions of FW zebrafish and rohu (*Labeo rohita*) spermatozoa [20,21] increases the duration of motility [20]. Amongst marine fishes, however, the functional contribution of ions and ion channels to the molecular regulation of spermatozoon swimming performance remain largely unknown.

In the present work we therefore investigated the potential function of ion channels in the activation and prolongation of sperm motility in the seabream, a commonly used model organism for modern marine teleosts. To achieve this, we followed three consecutive approaches. First, we established whether changes in Ca^2+^, K^+^ and Na^+^ and pH_i_ are required for the acquisition and maintenance of flagellar movement by using different extender and hypertonic activation media and pH conditions. Second, we examined the type of ion channels expressed in the seabream ejaculated spermatozoa through transcriptome meta-analysis and immunological methods. Finally, we investigated whether the pharmacological targeting of selected groups of ion channels can affect the motility, velocity, and swimming pattern, of post activated spermatozoa.

## 2. Results

### 2.1. A Surge of Intracellular Ca^2+^, but Not of K^+^ or Na^+^, Is Necessary for Sperm Motility Activation in the Seabream

**Figure 1:**
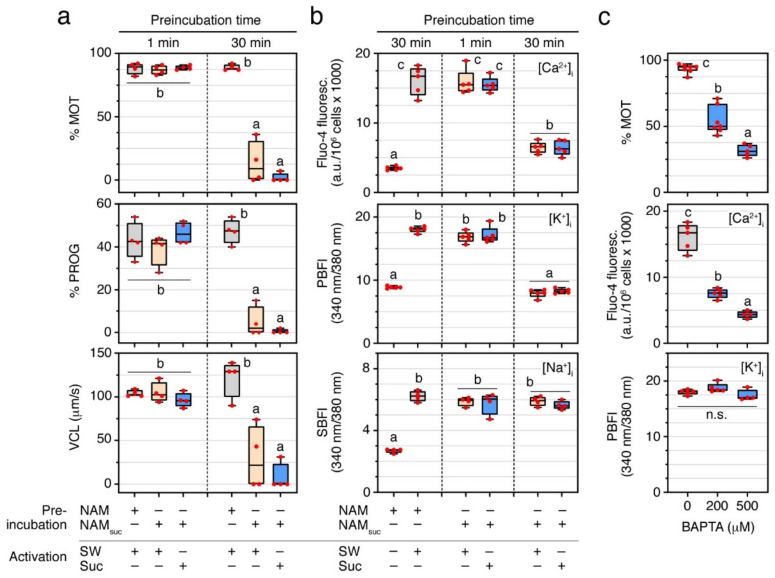
A surge of [Ca^2+^]_i_, but not [K^+^]_i_ or [Na^+^]_i_, independent of external ions, is necessary for the activation of sperm motility in the seabream. (**a**) Effect of different incubations times in standard non-activating medium (NAM) or NAM containing sucrose and no ions (NAM_suc_), and further activation in seawater (SW) or sucrose (Suc) for 5 s, on spermatozoa motility (% MOT), progressivity (% PROG), and curvilinear velocity (VCL). (**b**) Levels of [Ca^2+^]_i_, [K^+^]_i_ and [Na^+^] at 5 s post activation in spermatozoa treated as in (**a**). (**c**) Percentage of MOT, and intracellular levels of Ca^2+^ and K^+^, in sperm activated in SW containing increasing doses of BAPTA. In all panels, the data points are presented as box and whisker plots/scatter dots with horizontal line (inside box) indicating median and outliers. One ejaculate from each male was measured from *n* = 4–7 males. Data were statistically analyzed by one-way ANOVA. Boxes with different superscript are statistically significant (*p* < 0.05). n.s., not significant.

Previous studies of the seabream suggest that extracellular Ca^2+^ or K^+^ are not necessary for the activation of sperm motility [15,22]. To confirm these observations, and to investigate the role of external Na^+^ in this process, ejaculated and immotile seabream spermatozoa were incubated in normal non-activating medium (NAM), or in NAM containing sucrose and no ions (NAM_suc_) (Table 1), for 1 or 30 min, and subsequently activated by a hyperosmotic shock in SW or 1.1 M sucrose. Sperm motility assays using computer-assisted sperm analysis (CASA) showed that the percentage of total motile and progressive spermatozoa (% MOT and % PROG, respectively) and curvilinear velocity (VCL) at 5 s post activation was not affected (*p* > 0.05) by incubation in NAM_suc_ for 1 min, regardless of whether activation occurred in either SW or sucrose (Figure 1a). These observations thus corroborate that neither extracellular Ca^2+^, K^+^ nor Na^+^ are required for motility activation. However, when immotile spermatozoa were incubated in a non-ionic medium such as NAM_suc_ for 30 min prior to activation in SW or sucrose, the kinematic properties of activated spermatozoa were highly reduced (*p* < 0.05) with respect to those preincubated in NAM for the same period of time (Figure 1a).

To examine whether a 30 min incubation of ejaculated spermatozoa in NAM_suc_ can affect the intracellular ion levels before activation, we used Ca^2+^-, K^+^- and Na^+^-specific fluorescent acetoxymethyl (AM)-ester dyes. For this, immotile sperm was loaded with one of the dyes prior to their incubation in NAM_suc_ for 1 or 30 min, or in regular NAM for 30 min, and subsequently activated in SW or sucrose. Fluorometric quantification showed a 4.6-, 2.0-, and 2.3-fold increase (*p* < 0.05) of [Ca^2+^]_i_, [K^+^]_i_ and [Na^+^]_i_, respectively, at 5 s post activation in spermatozoa previously incubated with NAM for 30 min and activated in SW when compared to immotile spermatozoa (Figure 1b). A similar increase of [Ca^2+^]_i_, [K^+^]_i_ and [Na^+^]_i_ was observed in NAM_suc_-incubated spermatozoa for 1 min and activated in either SW or sucrose (Figure 1b). In contrast, incubation in NAM_suc_ for 30 min selectively induced a decrease (*p* < 0.05) of [Ca^2+^]_i_ and [K^+^]_i_, but not of [Na^+^]_i,_ in SW or sucrose activated spermatozoa (Figure 1b). These and previous data therefore indicate that an increase of [Ca^2+^]_i_ and/or [K^+^]_i_ is required for the initiation of motility of seabream spermatozoa, and that extended incubation times of immotile spermatozoa in non-ionic mediums possibly result in intracellular Ca^2+^ and K^+^ ion losses, which affect subsequent activation.

To further investigate which of the intracellular Ca^2+^ or K^+^ ions is essential for motility initiation, NAM-incubated immotile spermatozoa were activated in SW containing increasing concentrations (200 and 500 µM) of the intracellular Ca^2+^ chelator 1,2-bis(2-aminophenoxy)ethane-N,N,N′,N′-tetraacetic acid (BAPTA), and the [Ca^2+^]_i_ and [K^+^]_i_, as well as the motility properties of the spermatozoa, were then determined. Upon SW activation, treatment with BAPTA reduced the % MOT in a dose-dependent manner (*p* < 0.05) with respect to 0.5% dimethyl sulfoxide (DMSO)-treated spermatozoa (controls), which was accompanied by a significant (*p* < 0.05) decrease of the [Ca^2+^]_i_ levels, but not of those of [K^+^]_i_ (Figure 1c). Therefore, these findings suggest that an increase of only the [Ca^2+^]_i_ is necessary to initiate sperm motility in the seabream.

### 2.2. Basic pH Is Necessary to Activate and Maintain Sperm Motility 

**Figure 2:**
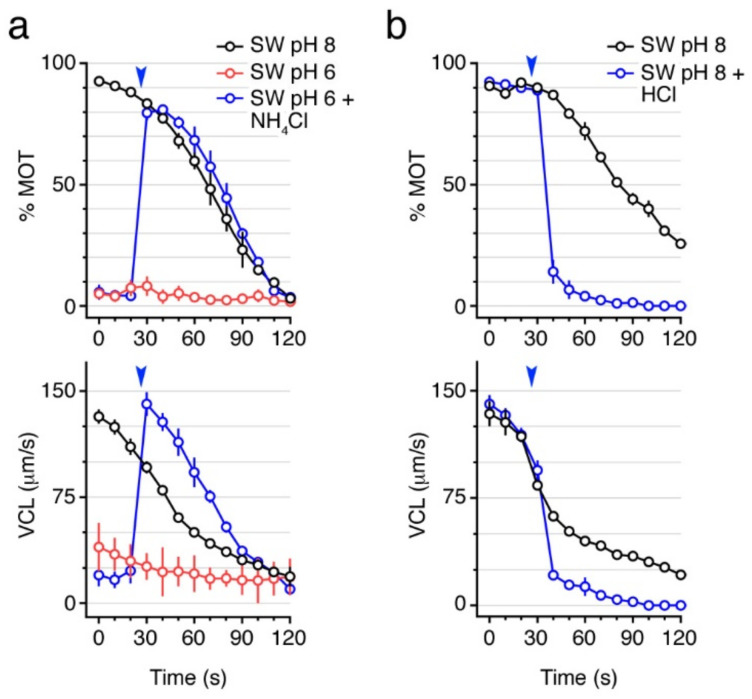
A basic pH is necessary to activate and maintain motility of seabream spermatozoa. (**a**) Time-course of total motile sperm (% MOT) and curvilinear velocity (VCL) upon activation in seawater (SW) at pH 6 or 8. Sperm activated at pH 6 was exposed to 250 mM NH_4_Cl at 25 s after activation (arrowhead). (**b**) % MOT and VCL of spermatozoa activated in SW at constant pH 8, or exposed to 1.6 mM HCl at 25 s after activation (arrowheads). Data are the mean ± SEM (*n* = 3–4 males, one ejaculate per male).

In both FW and SW teleosts, different studies have shown that pH_i_ is a key factor for the activation and maintenance of sperm motility, although the specific pH_i_ changes required for sperm motility seem to vary among species [12,23,24,25,26,27,28]. To investigate whether seabream sperm motility also depends on the pH_i_, we performed motility assays using SW at pH 6 or 8, which was alkalized or acidified by 250 mM NH_4_Cl or 1.6 mM HCl, respectively, at ~25 s post activation. Previous studies in the catadromous European eel have shown that the pH_i_ of spermatozoa is linearly dependent on the extracellular pH [12]. Our data showed that spermatozoa are not activated with SW at pH 6, whereas further intracellular alkalinization by NH_4_Cl triggers motility to the same extent as SW at pH 8 (Figure 2a). On the contrary, when spermatozoa are activated in SW at pH 8 and then exposed to 1.6 mM HCl to acidify the pH_i_, the % MOT and VCL were rapidly reduced (Figure 2b). These data suggest that a basic pH_i_ is necessary for the activation and maintenance of motility of seabream spermatozoa.

### 2.3. Ca^2+^ and pH_i_ Are Not Sufficient to Trigger Sperm Motility

**Figure 3:**
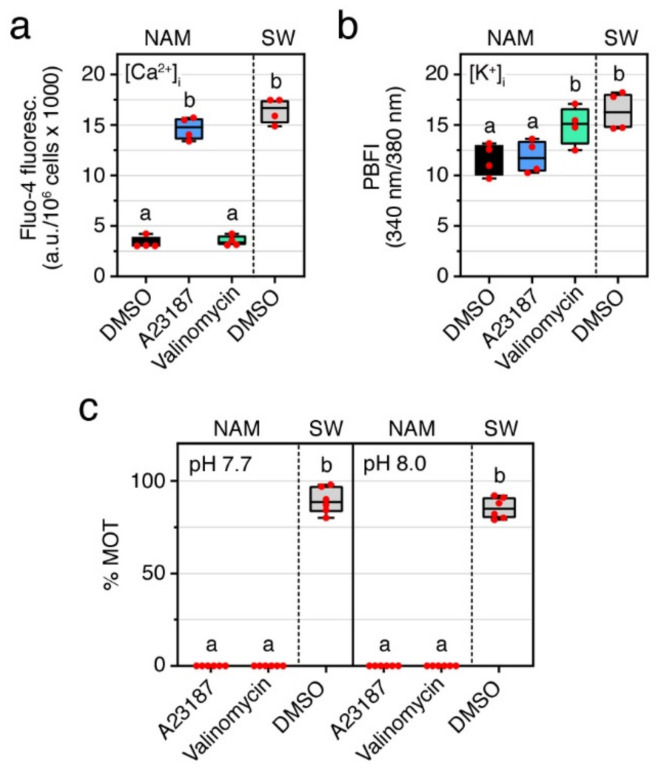
High levels of intracellular Ca^2+^ and basic pH are not sufficient for the activation of motility of seabream spermatozoa. (**a**,**b**) Intracellular Ca^2+^ (**a**) and K^+^ (**b**) levels in immotile sperm treated with 25 mM of the Ca^2+^ ionophore A23187 or the K^+^ ionophore valinomycin for up to 30 min, and in SW-activated spermatozoa. (**c**) Percentage of motility (% MOT) of the sperm treated as above under neutral and basic pH. In all panels, the data points are presented as box and whisker plots/scatter dots with horizontal line (inside box) indicating median and outliers. One ejaculate from each male was measured from *n* = 4–6 males. Statistical differences were measured by one-way ANOVA. Boxes with different superscript are statistically significant (*p* < 0.05).

The earlier experiments indicate that intracellular Ca^2+^ and alkaline pH_i_ are necessary for the activation of motility. To investigate whether these two conditions can trigger seabream sperm motility independently of the osmolarity of the external medium, as reported for some marine teleosts [29], spermatozoa were incubated in NAM at pH 7.7 or 8 containing 25 mM of the Ca^2+^ or K^+^ ionophores, A23187 and valinomycin, respectively, for up to 30 min and their motility evaluated up to 2 min. Fluorometric quantification showed that exposure of immotile spermatozoa to A23187 and valinomycin significantly increased (*p* < 0.05) the [Ca^2+^]_i_ and [K^+^]_i_ at ~5 s post activation to a similar levels than in SW-activated sperm (Figure 3a,b). However, the increment in [Ca^2+^]_i_ or [K^+^]_i_ did not induce activation of sperm motility at neither neutral nor basic external pH (Figure 3c). These observations suggest that the surge in [Ca^2+^]_i_ under a basic pH_i_ are necessary but not sufficient conditions to initiate sperm motility in seabream. Rather, these mechanisms also need to be associated with a hyperosmotic shock to trigger flagellar motility.

### 2.4. External Ca^2+^ and K^+^ Are Required for the Post Activation Maintenance of Sperm Motility

**Figure 4:**
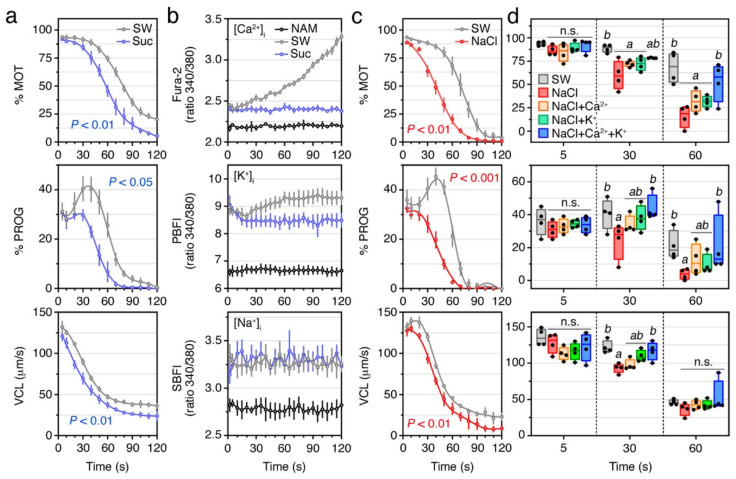
External ions are required for the maintenance of motility of post activated seabream spermatozoa. (**a**) Motility, progressivity, and VCL, of spermatozoa upon activation in SW or sucrose. (**b**) Intracellular Ca^2+^, K^+^ and Na^+^ levels in sperm activated in SW or sucrose. (**c**) Motility, progressivity, and VCL, of spermatozoa upon activation in SW or 550 mM NaCl. (**d**) Effect of activation in SW, NaCl, or NaCl, in which 10 mM Ca^2+^ and K^+^ were added separately or together, on seabream sperm kinetics. In a-c, data are the mean ± SEM (*n* = 4–5 males, one ejaculate per male), and were statistically analyzed by two-way ANOVA (*p* values indicated in each panel). In (**d**), data points (*n* = 4) are presented as box and whisker plots/scatter dots with horizontal line (inside box) indicating median and outliers. One ejaculate from each male was measured. Statistical differences were measured by one-way ANOVA for each post activation time point, followed by the Tukey’s multiple comparison test. Boxes with different superscript are statistically significant (*p* < 0.05). n.s., not significant.

To further explore the role of external ions during the maintenance of sperm motility, we measured the kinetics parameters and intracellular levels of Ca^2+^, K^+^ and Na^+^ of SW- and sucrose-activated spermatozoa (Figure 4). Time-course monitoring of sperm motion kinetics showed that although sucrose can trigger motility, the % MOT, % PROG, and VCL, decreased relatively faster over time (*p* < 0.01, *p* < 0.05, and *p* < 0.01, respectively) when compared with SW-activated sperm (Figure 4a). Intracellular ion measurements showed that upon SW activation, [Ca^2+^]_i_ and [K^+^]_i_ rapidly increased with respect to immotile spermatozoa, but while the [Ca^2+^]_i_ progressively accumulated until motility resumed, the [K^+^]_i_ transiently decreased within approximately the first 20 s post activation to gradually increase thereafter (Figure 4b). The [Na^+^]_i_ in spermatozoa also increased upon activation, but no apparent further changes were noted during the motility time (Figure 4b). Upon sucrose activation, however, the first increases of the [Ca^2+^]_i_, [K^+^]_i_ or [Na^+^]_i_ were not affected, but the further increments of [Ca^2+^]_i_ and [K^+^]_i_ during motility time observed in SW-treated sperm were completely abolished (Figure 4b). These data indicate that extracellular Ca^2+^ and/or K^+^ are necessary to preserve the motility of seabream spermatozoa after activation.

To investigate the relative role of Ca^2+^ and K^+^ for the maintenance of sperm function, we carried out further experiments in which spermatozoa were activated in 550 mM NaCl alone, or containing 10 mM CaCl_2_, 10 mM KCl, or both. As noted in previous studies of seabream [15,22], the hyperosmotic shock caused by NaCl activates sperm motility (Figure 4c). However, as observed in spermatozoa activated with sucrose, the % MOT, % PROG, and VCL, of NaCl-activated spermatozoa were not affected at 5 s post activation with respect to sperm activated in SW, but the motion parameters reduced more rapidly over time (*p* < 0.01, *p* < 0.001, and *p* < 0.01, respectively) when compared with the SW control spermatozoa (Figure 4c,d). Interestingly, the addition of 10 mM of Ca^2+^ and K^+^ to the NaCl activating medium restored the % MOT, % PROG, and VCL, of spermatozoa within 30 to 60 s post activation (Figure 4d). This indicates that both extracellular Ca^2+^ and K^+^, although not necessary for the activation of motility, are required to maintain the kinematic properties of the spermatozoon.

### 2.5. Seabream Spermatozoa Express Multiple Ion Channels 

**Figure 5:**
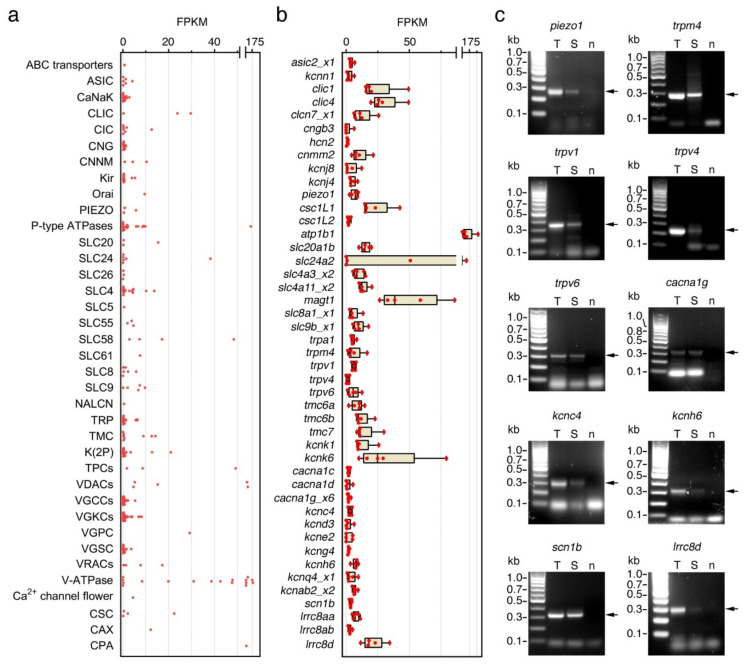
Ion channel-encoding genes expressed in ejaculated seabream spermatozoa. (**a**) Plot showing the mean expression levels of all the 342 genes assessed as fragments per kilo base per million mapped reads (FPKM) of the corresponding transcripts. (**b**) Levels of expression of the most abundant transcripts encoding for ion channels potentially localized in the plasma membrane of the spermatozoa determined in 5 replicate RNA-seq libraries of ejaculated spermatozoa (each replicate being a pool of cells collected from three different males). Data points are presented as box and whisker plots/scatter dots with horizontal line (inside box) indicating median and outliers. Data in a and b were calculated from a recent RNA-seq analysis on seabream spermatozoa reported by Castro-Arnau et al. [30] (Gene Expression Omnibus database accession no. GSE173088, National Center for Biotechnology Information). (**c**) Representative RT-PCR detection of mRNAs encoding selected ion channels in testis (T) and ejaculated spermatozoa (S). The n line is the negative control (absence of RT during cDNA synthesis). The arrows indicate the specific amplified transcripts, and the size (kb) of molecular markers are indicated on the left. Uncropped gels are shown in Supplementary Figure S2). See Spreadsheets S1 and S2 for abbreviations and gene names.

The results of the previous experiments suggest the presence of Ca^2+^ and K^+^ channels in seabream spermatozoa that may play a role in the maintenance of flagellar motility. To investigate this, we carried out a meta-analysis of the data from a recent RNA-seq transcriptomic study during seabream spermiogenesis [30] in order to identify which types of ion channels are potentially expressed in the ejaculated spermatozoon. The results of this analysis revealed that seabream spermatozoa express 342 ion channel-encoding genes belonging to 38 different families of ion channels, the groups of voltage-gated K^+^ channels (VGCCs) and voltage-gated Ca^2+^ channels (VGKCs) being the most represented (Figure 5a, Supplementary Spreadsheet S1). Most of the genes (297) encode for channels potentially localized in the plasma membrane, whereas the rest (45) may be expressed in the nuclear or mitochondrial membrane, endoplasmic reticulum, or intracellular vesicles (Supplementary Spreadsheet S1).

The top 10 expressed genes assessed as fragments per kilo base per million mapped reads (FPKM) of the corresponding transcripts with corresponding protein products that can be localized in the plasma membrane, encoded for Na^+^/K^+^-transporting ATPase subunit beta 1 (*atp1b1*; 148 FPKMs), magnesium transporter 1 (*magt1*; 48 FPKMs), Na^+^/K^+^/Ca^2+^ exchanger 2-like (*slc24A2*; 38 FPKMs), chloride intracellular channel 1 and 4 (*clic1* and *-4*; 23 and 29 FPKMs, respectively), voltage-gated H^+^ channel 1 (*hvcn1*; 29 FPKMs), Ca^2+^ permeable stress-gated cation channel 1-like protein 1 (*csc1l1*; 22 FPKMs), K^+^ channel subfamily K member 6 (*kcnk6*; 21 FPKMs), volume-regulated anion channel subunit Lrrc8d (*lrrc8d*; 17 FPKMs) and Na^+^-dependent phosphate transporter 1-B (*slc20a1b*; 15 FPKMs) (Figure 5b, Supplementary Spreadsheet S2). Other lower expressed genes encoding for plasma membrane ion channels were metal transporter Cnnm2 (*cnnm2*; 10 FPKMs), *trpv1*, *-4* and *-6* (6, 6 and 1 FPKMs, respectively), *trpa1* (6 FPKMs), *trpm4* (6 FPKMs), voltage-dependent L-type Ca^2+^ channel subunit alpha-1C, 1D and 1G (*cacna1c, -1d and -1g*; all with 2 FPKMs), piezo-type mechanosensitive ion channel component 1 (*piezo 1*; 6 FPKMs), K^+^ voltage-gated channel subfamily C member 4 and subfamily H member 6 (*kcnc4* and *kcnh6*; 3 and 2 FPKMs, respectively), and Na^+^ channel subunit beta-1 (*scn1b*; 4 FPKMs) (Figure 5b, Supplementary Spreadsheet S2). The expression of some of these transcripts (*piezo 1*, *trpm4*, *trpv4*, *trpv1*, *trpv6*, *cacna1g*, *kcnc4*, *kcnh6*, *scn1b* and *lrrc8d*) in ejaculated spermatozoa was confirmed by reverse-transcription PCR (RT-PCR) using gene specific oligonucleotide primers (Figure 5c). 

**Figure 6:**
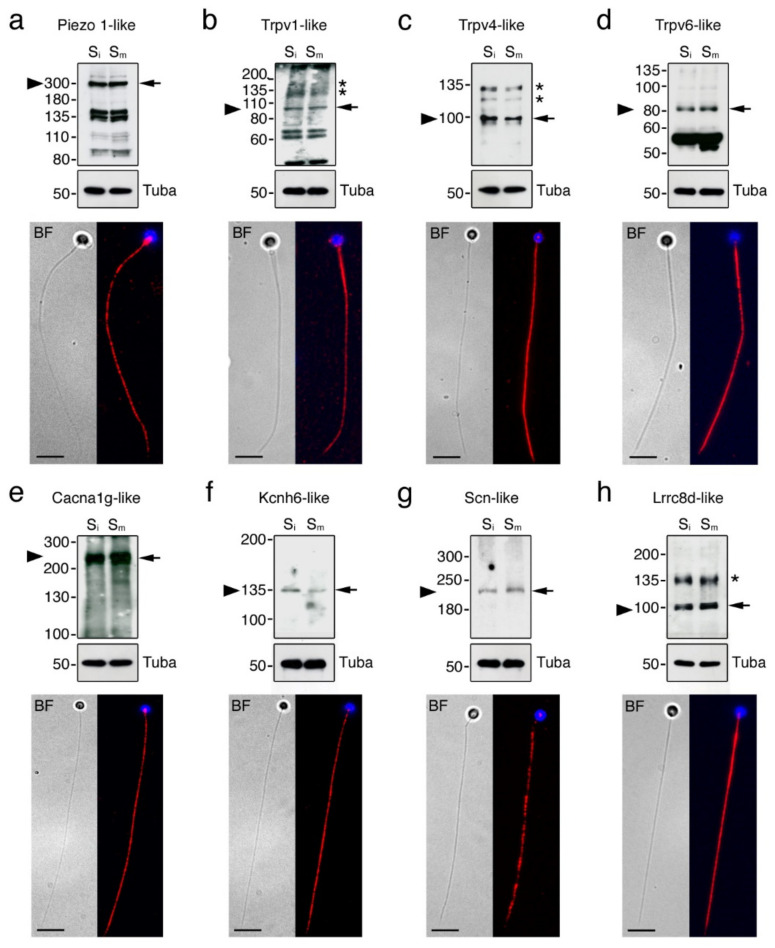
Confirmation of protein expression of different ion channels in seabream immotile and activated spermatozoa. (**a**–**h**, upper panels) Immunoblots of selected ion channels in immotile and motile spermatozoa (S_i_ and S_m_, respectively). Alpha-tubulin (Tuba) was used as a marker for even loading. Arrows indicate channel monomers and arrowheads the expected size of the target bands based on in silico determination of molecular masses. Asterisks indicate potential post translational modifications. Molecular mass markers (kDa) are on the left. Uncropped immunoblots are shown in Supplementary Figure S3. (**a**–**h**, lower panels) Representative bright field (BF) images (**left**) and immunodetection (**right**; red color) of ion channels in ejaculated immotile spermatozoa. The spermatozoon nucleus is counterstained with DAPI (blue). Scale bars, 5 µm.

To further assess the presence of the protein products of some of the ion channel mRNAs amplified by RT-PCR in spermatozoa, Western blot and immunofluorescence microscopy were carried out using commercial antibodies against the mammalian orthologs. In agreement with the mRNA data, Piezo 1-like, Trpv1-like, Trpv4-like, Trpv6-like, Cacna1g-like, Kcnh6-like, Scn-like and Lrrc8d-like immunoreactive bands were detected in both non-activated and activated spermatozoa by immunoblotting employing protein extracts from total sperm, sperm membranes or flagella (Figure 6a–h, upper panels). For Cacna1g, Kcnh6 and Scn single immunoreactive bands of approximately the same molecular mass as the predicted monomers were detected (Figure 6e–g, upper panels), whereas for the Trpv1 and -4 and Lrrc8d channels additional secondary bands of higher molecular masses than the predicted monomers were revealed (Figure 6b,c,h, upper panels), possibly corresponding to dimerization products and/or complex post translational modifications of the channels in spermatozoa. For Piezo 1, Trpv6, and also Trpv1, other bands of lower molecular masses than the monomers were noted (Figure 6a,b,d, upper panels), which could correspond to degraded protein products. For all the ion channels tested no apparent changes in the amount of the proteins were detected between non-activated and activated spermatozoa.

Immunostaining experiments revealed that all the ion channels investigated were distributed along the entire flagellum of immotile spermatozoa, except the Trpv1-like channels, which were also detected in the head (Figure 6a–h, lower panels). The Trpv1- and Lrrc8b-like channels were somewhat more accumulated in the anterior part of the tail (Figure 6b,h, lower panels), whereas Trpv4- and Kcnh6-like immunostainings appeared slightly more intense along the posterior region of the flagellum (Figure 6c,f, lower panels).

### 2.6. Ion Channel Blockers Affect the Motility of Post Activated Spermatozoa

**Figure 7:**
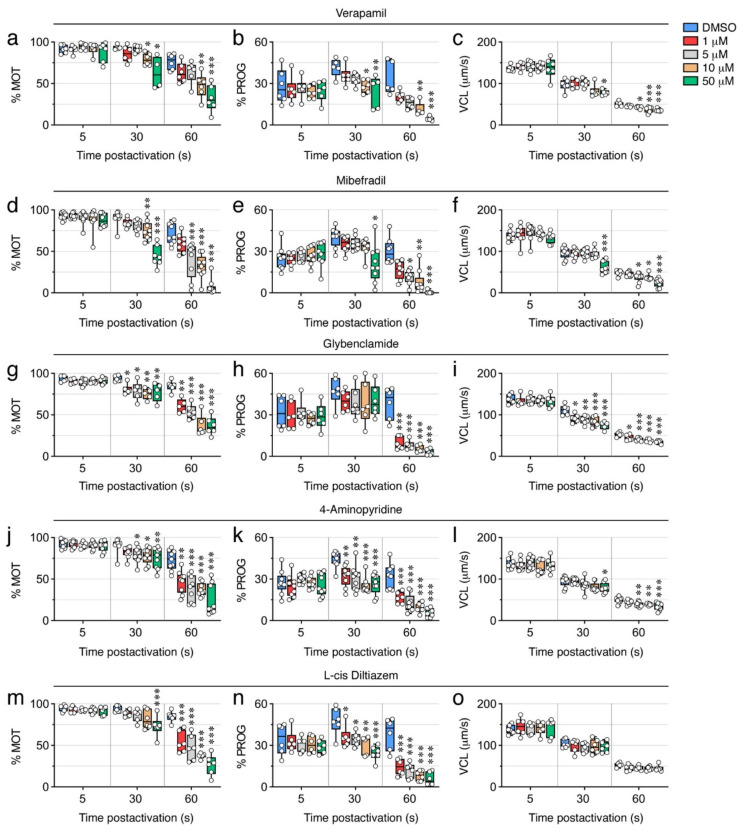
Inhibition of seabream sperm motility by blockers of Ca^2+^ channels, ATP-sensitive and voltage-dependent K^+^ channels, and CNG channels. (**a**–**o**) Dose-response inhibition of the percentage of motility and progressivity (% MOT and % PROG, respectively) and curvilinear velocity (VCL) at 5, 30 and 60 s post activation induced by the different ion channel blockers as indicated. Control spermatozoa were treated with 0.5% DMSO. In all panels, the data points (*n* = 6–8 males, one ejaculated per male) are presented as box and whisker plots/scatter dots with horizontal line (inside box) indicating median and outliers. Statistical differences were measured by one-way ANOVA (*, *p* < 0.05; **, *p* < 0.01; ***, *p* < 0.001, with respect to DMSO-treated sperm).

**Figure 8:**
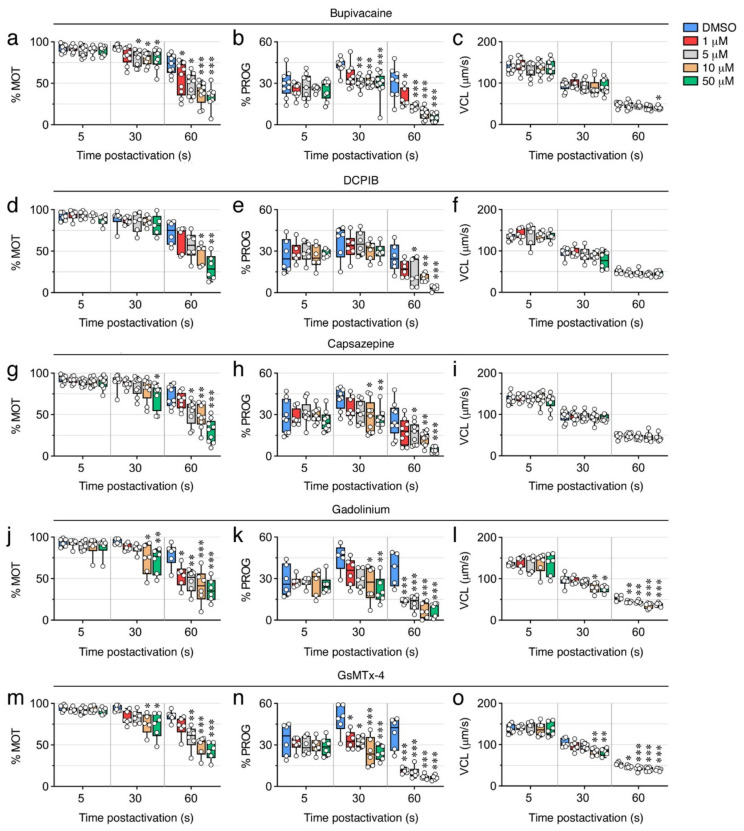
Inhibition of seabream sperm motility by blockers of voltage-gated Na+ channels, VRAC, TRPV, SACs, and MSCs. (**a**–**o**) Dose-response inhibition of the percentage of motility and progressivity (% MOT and % PROG, respectively) and curvilinear velocity (VCL) at 5, 30 and 60 s post activation induced by the different ion channel blockers as indicated. Control spermatozoa were treated with 0.5% DMSO. In all panels, the data points (*n* = 6–8 males, one ejaculated per male) are presented as box and whisker plots/scatter dots with horizontal line (inside box) indicating median and outliers. Statistical differences were measured by one-way ANOVA (*, *p* < 0.05; **, *p* < 0.01; ***, *p* < 0.001, with respect to DMSO-treated sperm).

A battery of different well-established ion channel antagonists (Table 2) was subsequently employed to investigate which ion channels may be involved in the maintenance of sperm motility. In these experiments, spermatozoa were preincubated in regular NAM and activated in SW containing increasing doses of the inhibitors (1, 5, 10, or 50 µM), or 0.5% DMSO vehicle, and the kinematic properties determined at 5, 30, and 60 s, post activation using CASA (Figure 7 and Figure 8). None of the inhibitors tested reduced the sperm motility at 5 s post activation, while inhibitory effects of most of the compounds were seen at 30 and 60 s post activation. 

Both of the Ca^2+^ channel blockers tested, verapamil and mibefradil, which are respectively selective for L-type and T-type Ca^2+^ channels, significantly inhibited (*p* < 0.05) the % MOT, % PROG, and VCL, at 30 s post activation at relatively high doses (10 and 50 µM) (Figure 7a–f). Mibefradil was somewhat more effective than verapamil at blocking sperm motility, since at 60 s post activation 5 µM mibefradil significantly inhibited the % MOT and % PROG by ~44% (*p* < 0.001) and ~62% (*p* < 0.05), respectively, whereas the same doses of verapamil were not effective (Figure 7a,b,d,e). However, both inhibitors at 5 µM reduced the VCL by ~20% (*p* < 0.05) at 60 s post activation (Figure 7c,f).

To evaluate the role of K^+^ channels, we used glybenclamide, which blocks ATP-sensitive K^+^ channels, and 4-aminopyridine, an inhibitor of voltage-dependent K^+^ channels. Both antagonists inhibited the % MOT in a dose-dependent manner at 30 and 60s post activation (*p* < 0.05), although 1 µM glybenclamide already blocked the % MOT at 30 s by ~14% (*p* < 0.05), while the inhibitory effect of 4-aminopyridine at this time was only noted with doses ≥5 µM (Figure 7g,j). However, while glybenclamide did not significantly affect the % PROG (*p* > 0.05) at 30 s up to a dose of 50 µM, 1 µM 4-aminopyridine was very effective, inhibiting the % PROG by ~27% (*p* < 0.01) (Figure 7h,k). At 60 s post activation, both drugs very efficiently blocked the % PROG in a dose-dependent manner (*p* < 0.001), reaching >80% inhibition at 50 µM (Figure 7h,k). The effect of the inhibitors on the VCL was different, since at 30 s post activation 1 µM glybenclamide already reduced the VCL by ~18% (*p* < 0.05), whereas 4-aminopyridine only inhibited the VCL by ~16% (*p* < 0.05) with 50 µM (Figure 7i,l). These data indicate that 4-aminopyridine was more effective than glybenclamide at inhibiting the progressivity of spermatozoa, whereas glybenclamide was more potent at blocking their velocity.

We next tested the effect of blockers of CNG channels, which can permeate both monovalent and divalent cations [31], and of voltage-gated Na^+^ channels. The CNG channel inhibitor L-cis diltiazem only reduced the % MOT by ~22% with 50 µM (*p* < 0.001) at 30 s post activation, whereas it was more efficient at 60 s post activation, when a dose of 1 and 50 µM inhibited the % MOT by ~33% and ~70%, respectively (*p* < 0.001) (Figure 7m). However, this inhibitor was more powerful at blocking the % PROG, since 1 µM significantly reduced the % PROG by ~26% (*p* < 0.05) and ~69% (*p* < 0.001) at 30 and 60 s, respectively (Figure 7n). The effect of the voltage-gated Na^+^ channel blocker bupivacaine on sperm motility was in general similar to that of L-cis diltiazem, except that bupivacaine was more effective at inhibiting the % MOT, showing a reduction of this parameter by ~14% (*p* < 0.05) with 5 µM at 30 s post activation (Figure 8a,b). The L-cis diltiazem did not significantly (*p* > 0.05) affect the VCL of the spermatozoa (Figure 7o), whereas bupivacaine only inhibited the VCL by ~16% (*p* < 0.05) with 50 µM at 60 s post activation (Figure 8c).

Pronounced volume changes of spermatozoa are likely to occur upon activation in the hyperosmotic SW. Therefore, volume-regulated anion channels (VRAC) and thermo- and osmotic-sensitive TRPV channels, both of them present in the seabream spermatozoon transcriptome, may play a role to preserve sperm motility. To investigate this, we further evaluated the effect on sperm motility of the VRAC blocker 4-(2-butyl-6,7-dichlor-2-cyclopentylindan-1-on-5-yl) oxobutyric acid (DCPIB) and the selective TRPV antagonist capsazepine. Our data show that DCPIB was less effective than the previous compounds, because significant (*p* < 0.05) reductions of the % MOT (by ~34%) and % PROG (by ~50%) by this drug were only seen with 10 µM and 5 µM, respectively, at 60 s post activation (Figure 8d,e). Capsazepine was slightly more potent, since this inhibitor could decrease (*p* < 0.05) the % MOT (by ~20%) and the % PROG (by ~34%) with 50 µM and 10 µM, respectively, at 30 s post activation (Figure 8g,h). However, neither DCPIB or capsazepine reduced the post activation VCL of spermatozoa even at the highest dose tested (Figure 8f,i).

Finally, to explore the role of cationic mechanosensitive channels (MSCs) and SACs during sperm motility, we tested gadolinium (Gd^3+^) and the spider venom peptide GsMTx-4. Both compounds significantly (*p* < 0.05) inhibited the % MOT by ~23–42% and ~25–52% with 10 µM and 50 µM, respectively, at 30 s post activation (Figure 8j,m). The effect of Gd^3+^ on the % MOT at 60 s post activation, however, was more efficient than that of GsMTx-4, since 1 µM Gd^3+^ induced an ~33% inhibition (*p* < 0.05), whereas GsMTx-4 only inhibited (by ~30%) at ≥5 µM (Figure 8j,m). In contrast, 1 µM of the GsMTx-4 antagonist blocked (*p* < 0.05) the % PROG by ~ 32% at 30 s post activation, whereas 1 or 5 µM of Gd^3+^ were ineffective during the same time (Figure 8k,n). The VCL of spermatozoa at 30 s post activation was equally reduced by ~20% (*p* < 0.05) with 10 µM of both inhibitors, whereas the two drugs were slightly more potent at blocking the VCL at 60 s post activation, from 13 to 30% inhibition with 1 to 50 µM (Figure 8l,o). These data suggest that MSCs may be more important than SACs to regulate the progressivity of spermatozoa, whereas both types of channels seem to regulate the motility and velocity of spermatozoa.

### 2.7. Ion Channels Can Control the Sperm Swimming Pattern 

**Figure 9:**
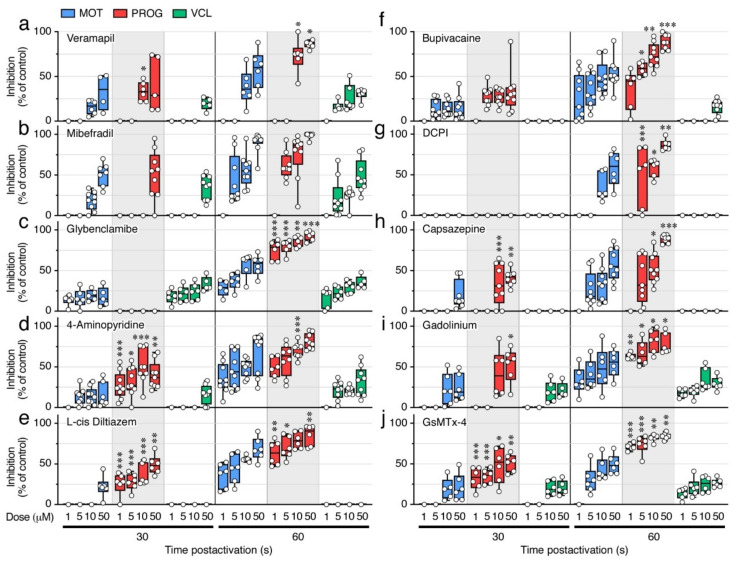
Percentage of inhibition of seabream sperm motility (MOT), progressivity (PROG), and curvilinear velocity (VCL), by the different ion channel blockers tested. (**a**–**j**) The percentage of inhibition at each dose and post activation time (30 or 60 s) with respect to DMSO-treated spermatozoa was calculated from data shown in Figure 7 and Figure 8. In all panels, the data points (*n* = 6–8 males, one ejaculated per male) are presented as box and whisker plots/scatter dots with horizontal line (inside box) indicating median and outliers. The asterisks indicate statistical differences between the percentage of inhibition of PROG and MOT at the same dose and post activation time (Student *t*-test; *, *p* < 0.05; **, *p* < 0.01; ***, *p* < 0.001).

The comparison of the percentage of inhibition of the % MOT and % PROG induced by each ion channel blocker indicated that some inhibitors, such as glybenclamide, 4-aminopyridine, L-cis diltiazem, capsazepine, Gd^3+^, and GsMTx-4, were more potent at reducing the % PROG than the % MOT at the same dose and post activation time (Figure 9). Glybenclamide did not affect the % PROG at 30 s post activation, but at 60 s 1 µM of this drug was ~3 times more potent (*p* < 0.001) at inhibiting the % PROG (75 ± 5%) than the % MOT (28 ± 4%) (Figure 9c). The 4-aminopyridine antagonist was able to block the % PROG by 27 ± 6% with 1 µM, whereas the same dose was not effective to reduce the % MOT (Figure 9d). A more evident effect was noted with L-cis diltiazem, which at 1, 5 and 10 µM during the first 30 s post activation inhibited the % PROG by 26 ± 6%, 28 ± 4% and 36 ± 5%, respectively, while the % MOT was only inhibited at doses ≥50 µM (Figure 9e). A similar observation was made for capsazepine, which reduced the % PROG by 34 ± 8% at 30 s post activation with 10 µM, while the % MOT was not affected (Figure 9h). The channel blocker Gd^3+^ had an effect similar to that of glybenclamide, since 1 µM inhibited the % PROG by 63 ± 2% at 60 s post activation, thus being ~2 times more potent (*p* < 0.01) than its inhibition of the % MOT (33 ± 7%) (Figure 9i). Finally, the GsMTx-4 was as efficient as L-cis diltiazem, since 1 and 5 µM of this peptide blocked the % PROG by 32 ± 5% and 35 ± 3% at 30 s post activation, respectively, whereas the % MOT was unchanged (Figure 9j). These observations suggest that ion channels sensitive to these antagonists are possibly more involved in the control of the pattern of spermatozoa movement than the motility or velocity.

**Figure 10:**
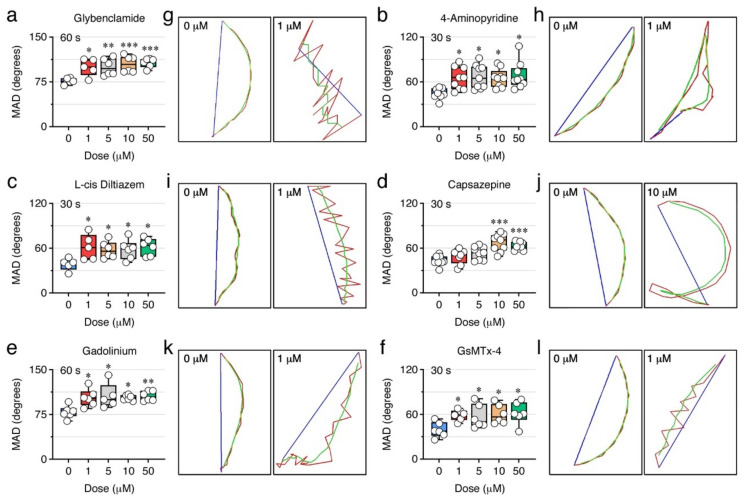
Effect of selected ion channel blockers on the trajectory of seabream spermatozoa. (**a**–**f**) Absolute mean angular displacement (MAD) of sperm treated with DMSO or the inhibitors at 30 or 60 s post activation in SW. Data points (*n* = 6–8 males, one ejaculated per male) are presented as box and whisker plots/scatter dots with horizontal line (inside box) indicating median and outliers. Statistical differences were measured by one-way ANOVA (*, *p* < 0.05; **, *p* < 0.01; ***, *p* < 0.001, with respect to DMSO-treated sperm). (**g**–**l**) Representative trajectory of a single activated spermatozoon in the presence of DMSO or the different inhibitors analyzed with ISAS^®^v1 CASA-Mot system. The red line shows curvilinear velocity (VCL), the blue line shows straight line velocity (VSL), and the green line shows average path velocity (VAP). The complete capture video sequences tracked at 25 frames per second are shown in Supplementary Figure S1.

To investigate this possibility, we further analyzed the mean angular displacement (MAD, degrees) of sperm treated with glybenclamide, 4-aminopyridine, L-cis diltiazem, capsazepine, Gd^3+^, or GsMTx-4, by using CASA. This kinematic parameter is defined as the time-averaged absolute values of the instantaneous turning angle of the sperm head along its curvilinear trajectory, and therefore is useful for evaluating the swimming trajectory of spermatozoa since it reflects the changes in their direction. The results of these analyses showed that all the ion channel blockers increased the MAD of spermatozoa in a dose-dependent manner (*p* < 0.05) (Figure 10). However, while 1 µM of 4-aminopyridine, L-cis diltiazem and GsMTx-4 significantly (*p* < 0.05) elevated the MAD already at 30 s post activation (Figure 10b,c,l), the same dose of glybenclamide and Gd^3+^ was only effective at 60 s post activation (Figure 10a,e). Capsazepine also increased the MAD at 30 s post activation, but only with a dose ≥ 10 µM (*p* < 0.001) (Figure 10d). The spermatozoa treated with the inhibitors showed in general curvilinear trajectories of lower curvature than the DMSO-treated spermatozoa (Figure 10g,h,i,j,k,l,), particularly for glybenclamide (Figure 10g), 4-aminopyridine (Figure 10h), L-cis diltiazem (Figure 10i), and GsMTx-4 (Figure 10l) (Supplementary Figure S1), thus revealing an altered wave motion pattern of these spermatozoa, which possibly affected their progression. These data therefore suggest that ATP-sensitive and voltage-gated K^+^ channels, CNG channels, SACs, and MCSs, may be involved in the control of the post activated swimming behavior of seabream spermatozoa.

## 3. Discussion

The present data show that extracellular Ca^2+^, K^+^ or Na^+^ ions are not involved in the activation of motility of seabream spermatozoa, thus confirming and extending previous observations [15,22]. On the contrary, the hyperosmotic shock-mediated increase of [Ca^2+^]_i_, together with the osmotic stress induced on the sperm cell and the alkalization of the cytosol, are the key mechanisms that need to concur simultaneously to initiate flagellar movement. Our data also indicate for the first time that the post activation motility and swimming performance of seabream spermatozoa are directly dependent on the presence of external ions and the function of different ion channels.

By using ion-specific fluorophores, we found that the [Ca^2+^]_i_, [K^+^]_i_ and [Na^+^]_i_ in spermatozoa increase upon activation independently of the presence of these ions in the external medium. These observations reinforce the notion that the rise of the ionic concentration in seabream spermatozoa mainly occurs via the massive water efflux mediated by flagellar Aqp1aa following the hyperosmotic activation, and/or, in the case of Ca^2+^, through the release of intracellular Ca^2+^ stores activated by Aqp1aa-mediated rapid cell shrinkage [15,22]. According to this model, we found that a 30 min incubation with an isosmotic non-ionic extender, such as sucrose, reduces the increase of the [Ca^2+^]_i_ and [K^+^]_i_ upon activation in either SW or sucrose, as well as the motility, progressivity, and velocity, of spermatozoa. Similar observations have been reported in Japanese and European eels (*Anguilla japonica* and *A. anguilla*, respectively) [32,33,34], suggesting that incubation of immotile spermatozoa with non-ionic mediums for relatively long periods of time may result in the loss of intracellular ions, thus affecting further activation. However, our experiments show, that although both the [Ca^2+^]_i_ and [K^+^]_i_ increase upon activation, the [K^+^]_i_ is not required to initiate motility, since the Ca^2+^-specific chelator BAPTA, which only reduced the [Ca^2+^]_i_ and not the [K^+^]_i_, was very effective to diminish the motility of spermatozoa. The increase of [Na^+^]_i_ upon activation does not seem to play a role on motility initiation either, as suggested for other fish species [29,35,36], since the long incubation of immotile spermatozoa with sucrose does not affect the further increase of the [Na^+^]_i_ upon activation, as observed for eel spermatozoa [35], while motility is markedly reduced. These observations therefore confirm previous studies of gilthead seabream [15,22] and striped seabream (*Lithognathus mormyrus*) [22] that suggest that only an increase of the [Ca^2+^]_i_ is required for the initiation of sperm motility, while neither K^+^ nor Ca^2+^ channels play a significant role.

In fish and mammals, it is known that the pH_i_ is crucial for the activation and maintenance of sperm motility [2,37]. Spermatozoa flagellar beating is controlled by pH sensitive axonemal dynein ATPases, which transduce chemical energy derived from ATP hydrolysis into the mechanical force necessary for the bending of flagella, and therefore the pH_i_ contributes to the regulation of flagellar motility [38,39]. Some studies have shown that the pH_i_ of fish spermatozoa varies in parallel with the extracellular pH (pH_e_) [12,27] and, in some fish species, the pH_e_ greatly affects the spermatozoon membrane potential regardless of the presence of Na^+^ or K^+^ in the external medium [40]. In many fish, as well as in mammals, alkalization of the pH_i_ is required for motility activation [2,12,37], although in some fish species, such as eels, sperm can become motile with acidification, or even with no change of the pH_i_ [12,26]. In our study, spermatozoa motility and velocity were completely abolished upon activation in SW at pH 6, whereas alkalization of SW at ~30 s post activation was able initiate flagellar motility. In addition, acidification of SW completely inhibited the post activated motility of seabream spermatozoa, as observed for other marine fishes [12,41,42,43]. Therefore, our data indicate that an alkaline pH_i_ is necessary for the activation and maintenance of the mechanical function of the axoneme in the seabream spermatozoa. 

In some marine species, such as the pufferfish (*Takifugu niphobles*) and flounder (*Kaireus bicoloratus*), it has been reported that introduction of Ca^2+^ into the sperm cells by a Ca^2+^ ionophore, as well as intracellular alkalization by treatment with ammonium salts, are each able to activate sperm motility under isosmotic conditions [29]. Further studies, however, showed that the increase in [Ca^2+^]_i_ itself had no significant effect on the motility and velocity of puffer fish sperm [44]. In addition, in salmonids a rise in pH_i_ is not sufficient to trigger spermatozoon motility [45]. Our data show that immotile seabream spermatozoa maintained in an isosmotic medium cannot be activated by a combined treatment with Ca^2+^ or K^+^ ionophores at basic pH_i_, indicating that a rise in [Ca^2+^]_i_ together with an alkaline pH_i_ need to concur with an hypertonicity-induced osmotic stress to trigger motility. The cellular mechanisms by which the hypertonic stress itself is necessary to trigger flagellar beating in marine spermatozoa are not yet known. The activation of hypertonicity-induced cation channels (HICCs) [46] or mechanosensitive SACs [44,47] might be the mechanisms involved. However, the SACs antagonists tested in our study (Gd^3+^ and GsMTx-4) did not block the initial activation of sperm motility. This has previously been observed in seabream spermatozoa with a 20 min preincubation time with Gd^3+^ prior to activation in SW [48]. Conversely, in other marine teleosts, such as sea bass (*Dicentrarchus labrax*), turbot (*Scophthalmus maximus*), tuna (*Thunnus thynnus*), and grass puffer (*Takifugu niphobles*), sperm activation is sensitive to Gd^3+^, suggesting that SACs are likely to participate in the initiation of flagellar movement [3,44,47,49]. Therefore, the type of cell volume-regulated ion channels that might be involved in the hypertonicity-triggered sperm motility initiation mechanism in seabream remains intriguing and needs to be investigated further.

The time-course monitoring of the motion kinetics of post activated seabream spermatozoa in SW, sucrose, or NaCl, as well as of the changes in the [Ca^2+^]_i_ and [K^+^]_i_, revealed that the spermatozoon velocity and progressive motility is, in contrast to the activation of motility, dependent on an influx of Ca^2+^ and K^+^ ions from the external medium. These findings therefore suggest the role of Ca^2+^ and K^+^ channels in the maintenance of post activated sperm motility. In support of this hypothesis, the transcriptome meta-analysis showed that the seabream ejaculated spermatozoa express a high number of VGCCs and VGKCs, as well as other channels that can also transport Ca^2+^ or K^+^ ions, such as TRP channels (Trpv1, -4, -6, Trpa1, Trmp4), Na^+^/Ca^2+^ exchangers, CNGK channels, SACs, or MSCs. We have shown that some of these channels are localized in the head and/or the flagellum of the seabream spermatozoon, and their mammalian and piscine orthologs have been shown to play a role in the control of the spermatozoon motility [4,5,20,21,33,44,49,50,51,52,53,54,55]. The potential role of some of these ion channels in the preservation of post activated sperm motility in seabream is supported by our pharmacological targeting experiments, where inhibitors of L- and T-type Ca^2+^ channels (verapamil and mibefradil, respectively), CNG channels (L-cis diltiazem), TRPV channels (capsazepine), SACs (Gd^3+^), and MSCs (GsMTx-4), as well as of ATP-sensitive K^+^ channels (glybenclamide) and VGKCs (4-aminopyridine), reduced the motility, progressivity, and velocity, of spermatozoa at 30 and/or 60 s post activation. The mechanisms for the potential activation of these channels once flagellar beating has been triggered are not known, but interestingly we observed that the [K^+^]_i_ in spermatozoa transiently dropped within approximately the first 20 s post activation to gradually increase thereafter. This observation may indicate that an early massive efflux of K^+^ hyperpolarizes the spermatozoon membrane, which triggers the opening of Ca^2+^ transporting channels, as in FW fish spermatozoa. This mechanism remains however hypothetical and should be investigated in the future.

The role of external Na^+^ in the maintenance of post activated spermatozoon motility in seabream remains intriguing. We observed that although the [Na^+^]_i_ in spermatozoa increases upon activation, as do the [Ca^2+^]_i_ and [K^+^]_i_, the [Na^+^]_i_ does not change significantly over time either in SW or sucrose. Since sucrose is a non-ionic medium, our data may suggest the absence of a flux of Na^+^ during post activated sperm motility in seabream. However, the voltage-dependent Na^+^-channel blocker bupivacaine was very effective at reducing the % MOT and % PROG at 30 and 60 s post activation, suggesting that Na^+^ channels may play a role in the maintenance of the flagellar movement. Seabream ejaculated spermatozoa express mRNAs encoding different Na^+^ channels, and accordingly we have detected the presence of Scn-like peptides along the spermatozoon tail, as it has been shown in mammals [56,57]. Therefore, it is plausible that an increase of the local permeability to Na^+^ through Na^+^ channels, which cannot be detected by fluorometric methods, can still occur in the spermatozoon to maintain motility. Further studies should be conducted to determine the physiological role of Na^+^ in the maintenance of sperm motility in the seabream, and to elucidate how the spermatozoon can sustain a high [Na^+^]_i_ despite the absence of this ion in the activation media.

The pharmacological blockage of ion channels showed that the antagonists tested had differential effects on the motion kinetics of post activated spermatozoa, suggesting that the ion channels targeted may play different physiological roles. Our data indicate that VGKCs, CNG channels, voltage-gated Na^+^ channels, VRACs, and TRPV channels, may be predominantly involved in the regulation of the spermatozoon progressive motility, while ATP-sensitive K^+^ channels may be more critical to control the velocity. Additionally, MSCs appear to be more relevant than SACs to regulate the progressivity of spermatozoa. These conclusions however should be taken with caution since the specificity and affinity of the different antagonists tested for the orthologous teleost ion channels are unknown. Nevertheless, we observed that the blockage of ATP-sensitive K^+^ channels, VGKCs, CNG channels, TRPV channels, SACs, and MSCs, was very effective at altering the trajectory of spermatozoa, suggesting that these ion channels are involved in the control of the swimming pattern of the post activated spermatozoon. Most of these channels can transport Ca^2+^, which is known to induce the switch from a linear to a circular spermatozoon trajectory during the sperm-egg chemotactic mechanism common to many species [39]. Accordingly, it has been shown that the Ca^2+^ influx triggered by the CNGK channel-mediated hyperpolarization in zebrafish spermatozoa generates a ‘spinning’-like swimming pattern that can presumably guide the sperm into the micropyle [5]. It is thus possible that CNG channels may also play a role controlling the pattern of movement of seabream spermatozoa. However, our data suggest that additional K^+^-selective and mechanosensitive ion channels, which have not been yet characterized in seabream or in any other marine fish, may also be involved. 

## 4. Materials and Methods

### 4.1. Animals and Semen Collection

Semen was collected from adult 2–3-years old seabream males, maintained at the facilities of the Institut de Ciències del Mar (CSIC, Barcelona, Spain) as previously described [58]. During the natural reproductive season (November–February), fish were sedated by immersion in SW with 500 ppm of 2-phenoxyethanol (Merck KGaA, Darmstadt, Germany, 77699), and ejaculated sperm was collected after a soft pressure to the abdominal area of the fish using a syringe located in the gonopore to avoid SW or urine contamination. 

### 4.2. Reagents and Antibodies

The ion channel blockers were purchased as follows: verapamil (Merck, V4629), mibefradil (Merck, M5441), glybenclamide (Merck, G0639), 4-aminopyridine (Merck, 275875), L-cis diltiazem (Merck, D2521), bupivacaine (Merck, B5274), DCPIB (Tocris, Bio-Techne R & D Systems, S.LU, Madrid, Spain,1540), capsazepine (Merck, C191), gadolinium (Tocris, 4741) and GsMTx-4 (Smartox Biotechnology, Saint-Egrève, France, 08GSM001). The antibodies used are listed in Supplementary Table S1. All other reagents were purchased from Merck unless indicated otherwise. 

### 4.3. Sperm Motility Assays

Freshly collected sperm was diluted 1:100 in NAM (Table 1) [58], and spermatozoa concentration was determined by CASA using the Integrated Semen Analysis System (ISAS^®^v1, Proiser, Valencia, Spain) software as previously described [58]. Sperm (10^9^ cells/mL) was then preincubated for 1 or 30 min in different NAM media (Table 1), depending on the experiment, and activated by 1:10 dilution in filtered SW (1100 mOsm/kg), 1.1 M sucrose (1100 mOsm/kg) or 550 mM NaCl (1100 mOsm/kg) for 2–5 s. In some experiments, sperm was activated in SW in the presence of increasing doses of BAPTA (200 or 500 µM), or in SW at pH 6 or 8, with further exposure to 250 mM NH_4_Cl or 1.6 mM HCl at ~25 s post activation, to alkalize or acidify the pH_i_, respectively. In other experiments, immotile spermatozoa were treated with 25 mM of the Ca^2+^ ionophore A23187 (Merck, C7522) or the K^+^ ionophore valinomycin (Merck, V0627) at pH 7.7 or 8 for up to 30 min. To test the effect of Ca^2+^ and K^+^ on the maintenance of sperm motility, sperm was incubated in NAM(-Ca^2+^-K^+^), NAM(-Ca^2+^), or NAM(-K^+^) (Table 1) for 1 min, and activated in SW, 1100 mOsm/kg NaCl, or in 1100 mOsm/kg NaCl containing 10 mM Ca^2+^ and K^+^ separately (540 mM NaCl + 10 mM CaCl_2_, or 540 mM NaCl + 10 mM KCl, respectively) or together (530 mM NaCl + 10 mM CaCl_2_ + 10 mM KCl). Finally, the effect of different ion channel blockers on sperm motility was determined by activating sperm (10^8^ cells/mL) with SW in the presence of increasing concentrations (1, 5, 10 or 50 µM) of the inhibitors. In all the trials using drugs, control sperm was treated with 0.5% DMSO, which previous studies have shown that does not affect the motility of seabream spermatozoa [15,59].

The sperm motility parameters considered in this study were the percentage of total motile and progressive spermatozoa, and VCL. Spermatozoa were considered immotile if their VCL was <10 μm/s (see below), and the progressive motility was defined as the percentage of spermatozoa swimming forward in an approximate straight line. The VCL was defined as the time/average velocity of a sperm head along its actual curvilinear trajectory. These parameters were recorded with the ISAS^®^v1 CASA at ~5 s post activation or every 10 s up to 2 min at room temperature. In some experiments, the absolute MAD and the trajectories of sperm were also recorded and analyzed by using the ISAS^®^v1 CASA-Mot system. All kinematic analyses were run in triplicate for each ejaculate. The settings of the CASA system used were: counting chamber ISAS R2C10, camera ISAS 782C, 25 frames per s, image resolution 768 × 576 pixels, magnification × 20 phase, particle area 0–30 μm, connectivity (connection of the sperm head tracks within different frames) of 14 μm, VCL > 10 μm/s, path straightness > 10%, linearity > 10%, wobble > 10%, max. velocity (for tracking) 500 μm/s, and average path velocity > 10 μm/s.

### 4.4. Measurement of Intracellular Ions

Intracellular Ca^2+^, K^+^ and Na^+^ levels in sperm were estimated using different fluorescent acetoxymethyl (AM)-ester dyes. The Fluo-4-AM (Invitrogen, Thermo Fisher Scientific Inc., Waltham, MA, USA, F14201) or Fura-2-AM (Invitrogen F1221) were used for Ca^2+^, the PBFI-AM (Molecular Probes, Eugene, OR, USA, P-1267) for K^+^, and the SBFI-AM (Molecular Probes S-1263) for Na^+^. Fluo-4-AM was employed for the measurement of [Ca^2+^]i at one time point, while Fura-2 was used to monitor [Ca^2+^]i changes over the motility time. The dye stocks were prepared at 1 mM in DMSO, and immediately mixed at equal volumes with 20% Pluronic^®^ F-127 in DMSO before use. The mix was then diluted 1:100 in NAM containing immotile sperm (10^9^ cells/mL) and incubated for 1 h (final concentration of 5 µM for the AM-ester dyes in 0.2% Pluronic^®^ F-127). The sperm was centrifuged at 200× *g* for 5 min and the pellet washed and resuspended with NAM or NAM_suc_ to obtain 10^9^ cells/mL. A 10-µL aliquot of sperm (10^7^ cells) loaded with each dye was transferred to a 96-wells white plate (Nunc™ F96 MicroWell™, Thermo Fisher Scientific Inc., Waltham, MA, USA) and treated with 90 µL of NAM or NAM_suc_ (immotile controls), SW or sucrose solutions just prior to read the absorbance with emission/excitation of 345/494 for Fluo-4, or dual excitation at 340/380 nm while monitoring emission at 500 nm for Fura-2, PBFI or SBFI, and every 3 s during 2 min using a Spark^®^ multimode microplate reader (Tecan, Männedorf, Switzerland). For all dyes, the background from unloaded sperm cells was subtracted and the results were expressed as arbitrary fluorescence units per 10^6^ cells for Fluo-4, or as the ratio of fluorescence obtained at 340 nm with respect to that at 380 nm for Fura-2, PBFI or SBFI.

### 4.5. Meta-Analysis of a Seabream Sperm Transcriptome

The transcripts from the seabream ejaculated spermatozoa transcriptome annotated as ion channels or transporters [30] were classified into different families according to the International Union of Basic and Clinical Pharmacology (IUPHAR)/British Pharmacological Society (BPS) Guide to PHARMACOLOGY (https://www.guidetopharmacology.org/; accessed on 1 January 2021) [60]. The putative subcellular localization of each channel was determined using the Uniprot database (https://www.uniprot.org/; accessed on 1 January 2021). The expression levels of each transcript were estimated as FPKMs using the RSEM v1.3.0 [61] software. The details of RNA-seq library construction, sequencing and annotation are provided in Castro-Arnau et al. [30].

### 4.6. RT-PCR Expression Analysis

Total RNA from testis and ejaculated spermatozoa was extracted using the Qiagen (Hilden, Germany) RNeasy Plus Minit Kit following the manufacturer’s instructions. The cDNA was synthesized from 76 ng to 1 µg of total RNA using the AccuScript High-Fidelity 1st Strand cDNA Synthesis Kit (Agilent, Santa Clara, CA, USA, 200820). The PCR reaction was performed using 1 µL of cDNA, 5 IU Taq DNA polymerase (Merck, 11418432001), and 0.2 M of forward and reverse primers specific for the selected genes (Supplementary Table S2). Reactions were amplified using an initial denaturing step for 2 min at 94 °C, followed by 35 cycles of 94 °C for 1 min, 60 °C for 1 min, and 72 °C for 2 min, ending with a final elongation at 72 °C for 7 min. PCR products were run on 1% agarose gels and photographed.

### 4.7. Biochemical Fractionation of Spermatozoa and Protein Extraction

#### 4.7.1. Total Sperm

Fresh ejaculated sperm was diluted at 10^10^ cells/mL in NAM and 20 µL were activated with 80 µL of SW or diluted with 80 µL of NAM. Then, spermatozoa were mixed in ice-cold 2× RIPA buffer containing 300 mM NaCl, 100 mM Tris-HCl, pH 8, 2% Triton X-100, 1% sodium deoxycholate, 2 mM EDTA, 2 mM EGTA, EDTA-free protease inhibitors (Roche, Basilea, Switzerland, 11836170001), 2 mM Na_3_VO_4_, 2 mM NaF, and 200 U of benzonase (Merck, 103773). Cells were dissociated with a glass dounce homogenizer, sonicated for 20 s at 20% amplitude using a Digital Sonifier^®^ S250D (Branson Ultrasonics Corp., Danbury, CT, USA), and centrifuged at 14,000× *g* for 10 min at 4 °C. The supernatant was mixed with 2 × Laemmli sample buffer containing 5% β-mercaptoethanol, heated at 95 °C for 15 min, deep frozen in liquid nitrogen, and stored at −80 °C.

#### 4.7.2. Total Membrane

Amounts of 3 × 10^9^ immotile or activated spermatozoa were processed for total membrane extraction following the protocol previously described by Chauvigné et al. [58]. The sperm pellet was homogenized in 20 mM Tris-HCl (pH 7.8), 3 mM MgCl_2_, 0.25 M sucrose, and protease inhibitors, and centrifuged at 1000× *g* for 20 min at 4 °C. The resulting supernatant was ultracentrifuged at 100,000× *g* for 45 min at 4 °C, and the pellet resuspended directly in 100 µL of Laemmli sample buffer containing 5% β-mercaptoethanol and processed as above.

#### 4.7.3. Isolation of Flagella 

Fresh samples of immotile or activated sperm (3 × 10^9^ cells), diluted 1:10 in 500 µL of NAM or SW, respectively, were subjected to mechanical separation of head and flagellum by passing the cell suspension 10 times through a capillary (0.5 mm diameter) attached to a 1-mL syringe. After verifying the proper separation of heads and flagella under a microscope, the homogenate was centrifuged at 5000× *g* for 15 min at 4 °C and the pellet resuspended in 400 µL of 1% NaCl. The suspension was then loaded on top of a sucrose gradient made in a 2-mL Eppendorf tube (400 µL of 2 M, 1.5 M, 1 M, and 0.5 M sucrose, from bottom to top), and centrifuged at 21,000× *g* for 60 min at 4 °C. Flagella were recovered from the top of the gradient, diluted 4× with 1% NaCl and centrifuged at 10,000× *g* for 20 min at 4 °C. The resulting pellet was resuspended in 100 µL of Laemmli sample buffer containing 5% β-mercaptoethanol and processed as above.

### 4.8. Immunoblotting

Protein extracts obtained as indicated above were denatured at 95 °C for 10 min, electrophoresed in 7–10% SDS-PAGE and blotted onto nitrocellulose membranes for 3 h at 80 V. The membranes were blocked with either 5% nonfat dry milk or 3% bovine serum albumin (BSA) diluted in TBST (20 mM Tris, 140 mM NaCl, 0.1% Tween, pH 7.6), and incubated overnight at 4 °C, with specific primary antibodies (Supplementary Table S1). Bound antibodies were detected with horseradish peroxidase (HRP)-coupled anti-rabbit or anti-mouse IgG antibodies (Supplementary Table S1) for 1 h at room temperature. After washing in TBST, immunoreactive bands were revealed by the Immobilon^TM^ Western chemiluminescent HRP substrate (Millipore, Burlington, MA, USA, WBKLS).

### 4.9. Immunofluorescence Microscopy

Immotile ejaculated spermatozoa were attached to UltraStick/UltraFrost Adhesion slides (Electron Microscopy Sciences, Hatfield, PA, USA), activated in SW or NAM for 2 min and directly fixed on the slide in 4% paraformaldehyde in PBS (137 mM NaCl, 2.7 mM KCl, 100 mM Na_2_HPO_4_, 2 mM KH_2_PO_4_, pH 7.4) for 15 min. Different antigen retrieval protocols were then applied depending on the primary antibody employed (Supplementary Table S1). After blocking for 1 h in PBST (PBS with 0.1% Tween-20) containing 5% normal goat serum and 0.1% BSA, sections were incubated with primary antibodies (Supplementary Table S1) overnight at 4 °C in a humidified chamber. Anti-mouse or anti-rabbit secondary antibodies were applied for 1 h at room temperature at 1:800, subsequently cells were counterstained with 4′,6-diamidino-2-phenylindole dihydrochloride (DAPI; 1:3000; Merck, G8294) for 3 min in PBS to stain the nuclei. The sections were mounted with fluoromount aqueous anti-fading medium (Merck, F4680), and images were acquired at 100× magnification with a Zeiss Axio Imager Z1/ApoTome fluorescence microscope (Carl Zeiss Corp., Oberkochen, Germany). 

### 4.10. Statistical Analyses

Comparisons between two independent groups were made by the two-tailed unpaired Student’s *t*-test. The statistical significance among multiple groups was analyzed by one-way ANOVA, followed by the Tukey’s or Dunnett’s multiple comparison tests, or by the non-parametric Kruskal-Wallis test and further Dunn’s test for nonparametric post hoc comparisons, as appropriate. Time-course curves of sperm kinetic parameters were compared by the Mann-Whitney U test. Percentages were square root transformed prior to analyses. Statistical analyses were carried out using GraphPad Prism v9.4.1 (681) software (GraphPad Software). In all cases, statistical significance was defined as *p* < 0.05 (*), *p* < 0.01 (**), or *p* < 0.001 (***).

## 5. Conclusions

The present work confirms that the initiation of motility of seabream spermatozoa upon activation in SW requires an increase of the [Ca^2+^]_i_, alkalization of the cytosol, as well as a hyperosmotic shock. All three processes have to occur simultaneously to trigger flagellar beating. However, while the induction of motility is independent of extracellular ion fluxes, the long-term dynamics of intracellular ion concentration in ionic and nonionic activating media, as well as the pharmacological treatment of activated sperm, show that the post activated maintenance of spermatozoon swimming performance requires extracellular Ca^2+^ and K^+^ and the participation of different ion channels. Our data further reveal that Ca^2+^ transporting channels, such as CNG and TRPV channels, SACs and MSCs, as well as K^+^-selective channels, are involved in the regulation of the swimming trajectory of the spermatozoa, suggesting that these ion channels influence the pattern of movement of the post activated spermatozoon. These findings provide new insight into the signaling pathways regulating spermatozoon activation and swimming performance that are important for fertilization success in marine fishes.

## Figures and Tables

**Table 1:** Composition of the different non-activating mediums (NAM) employed in this study (in mM).

Compound.	NAM	NAM_suc_	NAM(-Ca^2+^,-K^+^)	NAM(-Ca^2+^)	NAM(-K^+^)
NaCl	75	-	81.5	80	75.6
KCl	1.5	-	-	1.5	-
MgCl_2_	12.9	-	12.9	12.9	12.9
CaCl_2_	2.65	-	-	-	2.65
NaHCO_3_	20	-	20	20	20
Glucose	4.4	-	4.4	4.4.	4.4
Bovine serum albumin	0.015	0.015	0.015	0.015	0.015
Sucrose	-	280	-	-	-
pH	7.7	7.7	7.7	7.7	7.7
mOsm/kg	280	280	280	280	280

Ejaculated sperm was diluted in one of these mediums for 1 or 30 min before activation in SW, 1.1 M sucrose or 530–550 mM NaCl.

**Table 2:** List of the ion channel blockers tested on seabream sperm kinetic parameters.

Compound	Selectivity
Verapamil	High voltage-activated (L-type) Ca^2+^ channels.
Mibefradil	Transient, low-voltage-activated (T-type) Ca^2+^ channels.
Glybenclamide	ATP-sensitive K^+^ channels.
4-Aminopyridine	Voltage-dependent K^+^ channels.
L-cis Diltiazem	Cyclic nucleotide-gated channels (nonselective monovalent and divalent cations).
Bupivacaine	Voltage-gated Na^+^ channels.
DCPIB	Volume-regulated anion channels (VRAC) (transport of Cl^−^, taurine, glutamate).
Capsazepine	Transient receptor potential vanilloid (TRPV) channels (highly Ca^2+^ selective).
Gadolinium	Stretch-activated ion channels (SACs) (Ca^2+^, Na^+^ and K^+^ transport).
GsMTx-4	Cationic mechanosensitive channels (MSCs) and SACs (Ca^2+^, Na^+^ and K^+^ transport).

## Data Availability

The seabream RNA-seq dataset analyzed in this study is available at the Gene Expression Omnibus (GEO) database from the National Center for Biotechnology Information (NCBI) under accession no. GSE173088 [30]. All other data are available from the corresponding author upon request.

## Supplementary Materials

The following supporting information can be downloaded at: https://www.mdpi.com/article/10.3390/ijms232012113/s1.
