# Supplementary material for: Role of Ion Channels in the Maintenance of Sperm Motility and Swimming Behavior in a Marine Teleost
Source: Int J Mol Sci. 2022 Oct 11;23(20):12113. doi: 10.3390/ijms232012113 (PMC9603624; doi:10.3390/ijms232012113)
Supplement: Supplementary file 1 [file ijms-23-12113-s001.zip › Supp Figures.pdf]

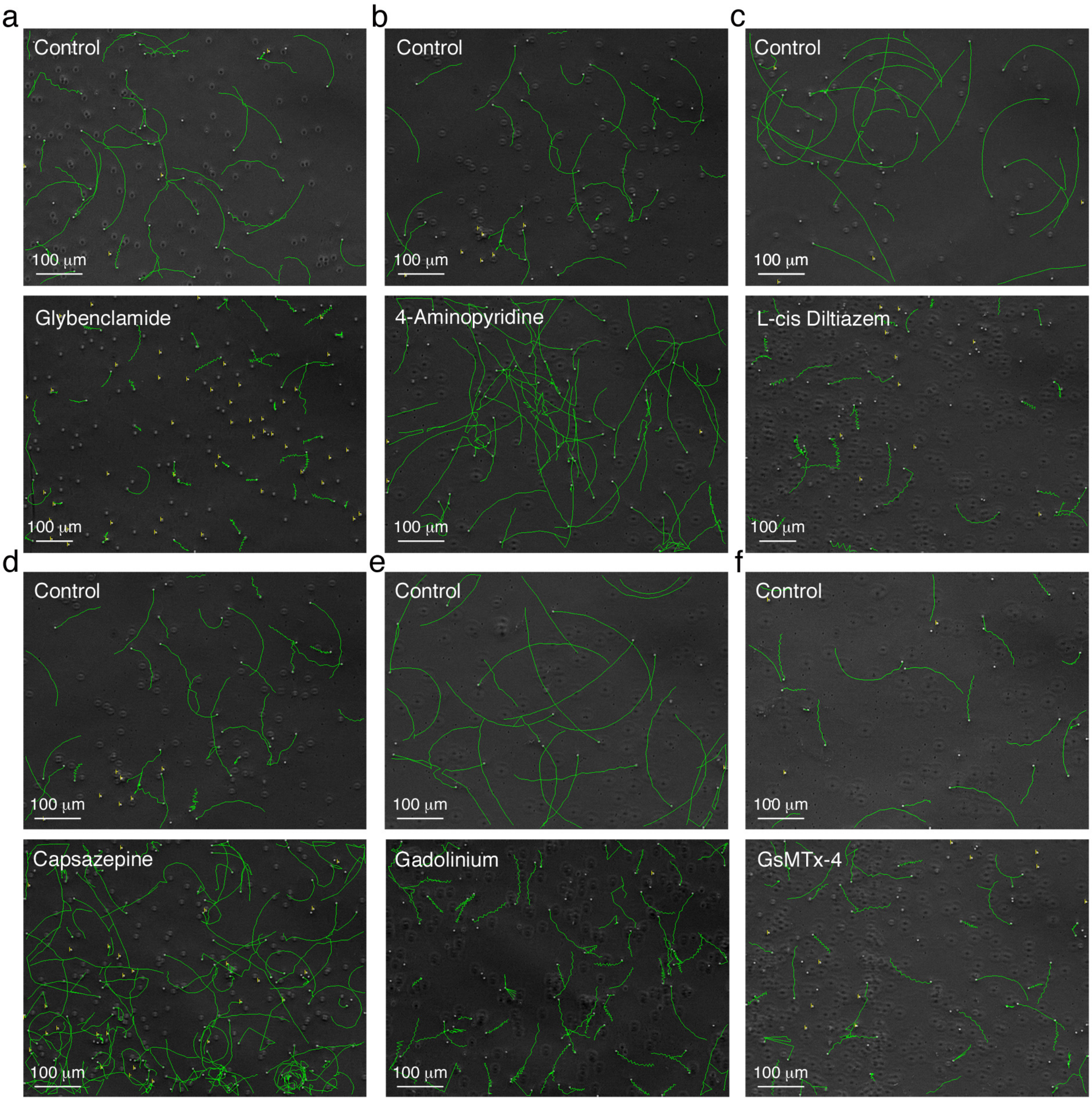


**Figure S1.** Representative sperm trajectories tracked at 25 frames per second in the presence of different ion channel blockers analyzed with ISAS®v1 CASA. The different types of motility are shown as trajectories of green color on the captured video sequences.

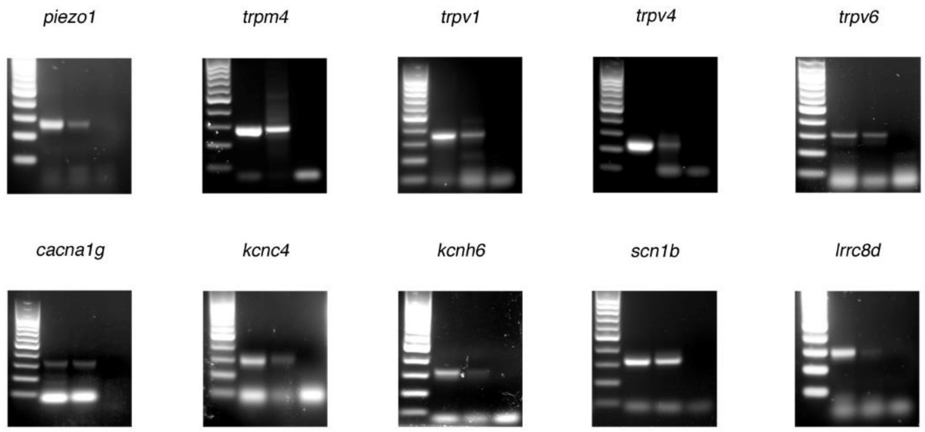


**Figure S2.** Uncropped RT-PCR gels shown in Figure 5c.

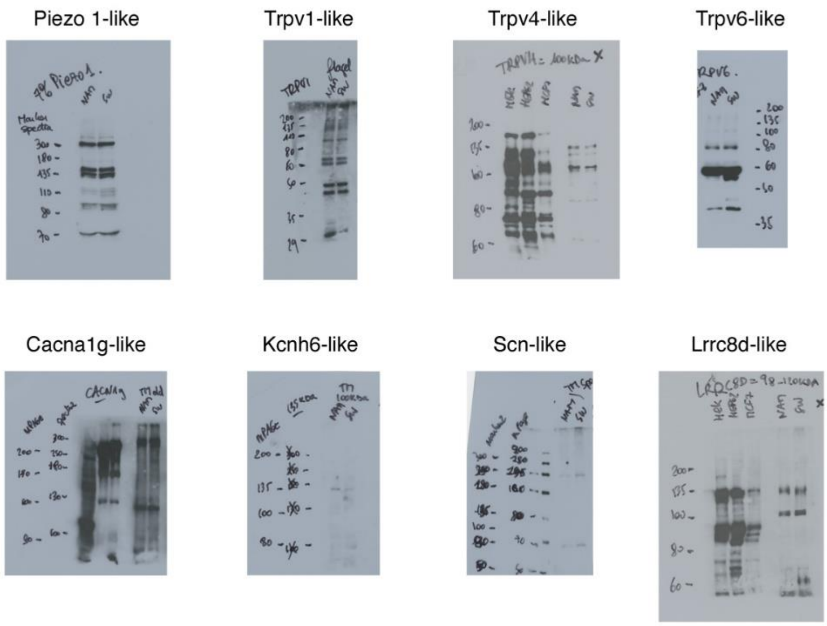


**Figure S3.** Uncropped immunoblots shown in Figure 6 (upper panels).
