# Supplementary material for: Role of Ion Channels in the Maintenance of Sperm Motility and Swimming Behavior in a Marine Teleost
Source: Int J Mol Sci. 2022 Oct 11;23(20):12113. doi: 10.3390/ijms232012113 (PMC9603624; doi:10.3390/ijms232012113)
Supplement: Supplementary file 1 [file ijms-23-12113-s001.zip › Supp Tables S1-S2.pdf]

Table S1. Primary and secondary antibodies employed in this study.

Antibody	Host species	Vendor	Catalog no.	Antigen[a]	% Identity[b]	Dilution[c] IB	Dilution[c] IF	Antigen retrieval[f]
PIEZO 1	Rabbit	Proteintech	15939-1-AP	Human 67-373 aa	56	1:250[d]	1:200	TE pH 9, 0.2% Triton X-100
TRPV1	Rabbit	Antibodies-online	ABIN351235	N/A	N/A	1:500[d]	1:400	0.2% Triton X-100
TRPV4	Rabbit	Novus Biologicals	NBP2-41262	Human 380-430 aa	78	1:500[d]	1:400	0.2% Triton X-100
TRPV6	Rabbit	Merck	SAB2106366	Mouse 838-987 aa	65	1:250[d]	1:400	0.2% Triton X-100
CACNA1G	Rabbit	GeneTex	GTX60133	Human 889-939 aa	96	1:250[e]	1:200	Citrate pH 6
KCNH6	Rabbit	Merck	AV35480	Human 1779-1928 aa	84	1:250[d]	1:200	Citrate pH 6
SCN (Pan antibody)	Mouse	Merck	S8809	Human 4979-5032 aa	100	1:200[e]	1:100	TE pH 9, 0.2% Triton X-100
LRRC8D	Rabbit	Proteintech	11537-1-AP	Human 515-858 aa	93	1:500[d]	1:200	TE pH 9, 0.2% Triton X-100
α-Tubulin	Mouse	Merck	T9026	Chicken microtubules	N/A	1:25000[d]	-	-
Rabbit IgG HRP	Goat	Bio-Rad	172-1019	-	-	1:5000[d]	-	-
Mouse IgG HRP	Goat	Bio-Rad	172-1011	-	-	1:5000[d]	-	-
Rabbit IgG Cy3	Sheep	Merck	C2306	-	-	-	1:1000	-

[a] Exact amino acid sequence of the mammalian ortholog used for the synthetic peptide employed for immunization, or amino acid region where the peptide was located (as available from the vendor).
[b] Percentage of identity of the immunization peptide or amino acid region with the seabream ortholog.
[c] IB: immunoblotting; IF: immunofluorescence microscopy.
[d] Blocking in 5% milk.
[e] Blocking in 3% BSA.
[f] Buffer employed for antigen retrieval prior to immunostaining.

Table S2. Nucleotide sequences of the primers employed for RT-PCR analysis of ion channel expression in seabream spermatozoa.

GenBank accession	Transcript symbol	Forward sequence	Reverse sequence
XM_030441522	*piezo1*	TGAGATCTCCCACTCCATCA	CCCCAAGACATCTTTGCAGT
XM_030400112	*trpm4*	GGCTTTGGAGAGTGAGATGG	GGTAACTGAGTCCAGCGATGA
XM_030438142	*trpv1*	GGAGAAGAGACTGGGCACTG	CGTAGGTTTGAATGCCTCGT
XM_030415727	*trpv4*	AGGGTGGATGAGGTGAACTG	TATCAGTGTCTGGGCGTCAG
XM_030393613	*trpv6*	GACAGAAACGACCCGATGTT	TTCAAATGTTCACGCCTCAG
XM_030399309	*cacna1g*	GAGCCTCAGTCTGGCCTCTA	TTTGTGTGTGTGGAGGGTTC
XM_030422644	*kcnc4*	CAAATGTGGCCCTTTCAGAG	CTGCACAGCGTCTACTCCAC
XM_030401465	*kcnh6*	GGTCACCCCTGAAACTGAGA	ACCTGAATCGCAGATTGGTC
XM_030411011	*scn1b*	CCGAGGTCATGATGTACGTG	TCCAGTTGTTGTCGAGGAGA
XM_030403786	*lrrc8d*	GTTCAGATGCACCAAGCTCA	TAAAGGGTGCGTTGCTCTCT
